# Elevated serum fibroblast growth factor 23 levels as an indicator of lower extremity atherosclerotic disease in Chinese patients with type 2 diabetes mellitus

**DOI:** 10.1186/s12933-017-0559-x

**Published:** 2017-06-15

**Authors:** Xingxing He, Xiang Hu, Xiaojing Ma, Hang Su, Lingwen Ying, Jiahui Peng, Xiaoping Pan, Yuqian Bao, Jian Zhou, Weiping Jia

**Affiliations:** Department of Endocrinology and Metabolism, Shanghai Jiao Tong University Affiliated Sixth People’s Hospital, Shanghai Clinical Center for Diabetes, Shanghai Key Clinical Center for Metabolic Disease, Shanghai Diabetes Institute, Shanghai Key Laboratory of Diabetes Mellitus, 600 Yishan Road, Shanghai, 200233 China

**Keywords:** Type 2 diabetes mellitus, Lower extremity atherosclerotic disease, Fibroblast growth factor 23, Femoral intima-media thickness

## Abstract

**Background:**

Recently, basic and clinical studies have provided evidence supporting the relationship between circulating levels of fibroblast growth factor (FGF) 23 and the development of atherosclerosis. Given that diabetes is an established risk factor for lower extremity atherosclerotic disease (LEAD), the goal of the present study was to explore the relationship between serum FGF23 levels and LEAD, as well as the related factors, in Chinese patients with type 2 diabetes mellitus (T2DM).

**Methods:**

A total of 401 hospitalized T2DM patients (201 subjects with LEAD and 200 subjects without LEAD) were enrolled in this study. Serum FGF23 levels were determined by a sandwich enzyme-linked immunosorbent assay. Femoral intima-media thickness (F-IMT) and lower limb atherosclerotic plaque were assessed through color Doppler ultrasound.

**Results:**

The median (interquartile range) serum FGF23 levels in the entire study population was 42.08 (35.59–49.17) pg/mL. Subjects with LEAD had significantly higher serum FGF23 levels compared with those without LEAD (44.00 [37.54–51.30] pg/mL versus 40.42 [32.61–48.23] pg/mL, *P* < 0.001). Logistic regression showed that serum FGF23 levels were independently and positively correlated with the presence of LEAD (odds ratio 1.039, 95% confidence interval 1.012–1.067, *P* = 0.004). In addition, multiple liner regression analysis revealed that serum FGF23 levels were positively associated with F-IMT (standardized *β* = 0.175, *P* < 0.001). Furthermore, this relationship remained significant after additional adjustment for gender and factors potentially affecting serum FGF23 levels (serum calcium, serum phosphorus, and glomerular filtration rate), respectively (both *P* < 0.01).

**Conclusions:**

In Chinese patients with T2DM, serum FGF23 levels were independently and positively correlated with the presence of LEAD.

## Background

Lower extremity atherosclerotic disease (LEAD), a primary manifestation of peripheral arterial disease (PAD), represents systemic atherosclerosis involving the peripheral vascular [1] and thus, is associated with an increased risk of cardiovascular disease [2, 3]. Diabetes is an established risk factor for LEAD [4], and the prognosis of LEAD in patients with diabetes is worse than that for those without diabetes, with a more rapid progression, wider range of vasculature lesions, and greater susceptibility to stenosis and occlusion [5]. In diabetes patients, given the common concurrence of neuropathy, LEAD often remains clinically undetected in the early stage. Diabetes patients are often unaware of the early progression of LEAD due to the loss of pain sensation and lower frequency of intermittent claudication until the symptoms become aggravated and advance to ulceration or gangrene, which are usually presented at the terminal stage of LEAD, leading ultimately to amputation [6]. Therefore, an indicator of early-stage LEAD is needed for the earlier diagnosis and treatment of this disease in patients with diabetes, which will help to avoid amputation and improve patients’ quality of life.

Fibroblast growth factor (FGF) 23, a protein mainly expressed by osteocytes and osteoblasts in bone, plays an important role in the regulation of mineral metabolism (calcium–phosphate homeostasis). Basic research studies have demonstrated that FGF23 induces the onset and development of atherosclerosis through its effects on vascular calcification and endothelial dysfunction [7, 8]. Recently, clinical studies provided evidence supporting the relationship between increased circulating FGF23 levels and atherosclerotic cardiovascular disease [9, 10]. Our previous studies revealed that serum FGF23 levels were associated with not only subclinical atherosclerosis but also coronary artery disease [11–13], suggesting the involvement of FGF23 in the onset and progression of atherosclerosis. In addition, we have also found that serum FGF23 levels are closely associated with the presence of abdominal obesity [14]. However, the association between serum FGF23 levels and LEAD remains to be determined in a Chinese population.

Color Doppler ultrasound shows advantages in the visualization of intima-media thickness (IMT) and plaque, and therefore, is widely used in the clinical setting for noninvasive assessment of PAD (especially LEAD) in patients with diabetes [15]. Hence, color Doppler ultrasound imaging was used to detect abnormalities in the lower extremity arteries in the present study, in order to investigate the relationship between serum FGF23 levels and LEAD, as well as the related factors, in Chinese patients with type 2 diabetes mellitus (T2DM).

## Methods

### Study population

Between March 2015 and December 2016, 401 patients (203 men, 198 women, age range: 25–78 years) with T2DM who were hospitalized in the Department of Endocrinology and Metabolism of Shanghai Jiao Tong University Affiliated Sixth People’s Hospital were enrolled in the present study. Patients with type 1 diabetes mellitus, gestational diabetes, or other specific types of diabetes were excluded. Additionally, individuals with hepatic dysfunction (acute vital hepatitis; alanine aminotransferase, or aspartate aminotransferase >1.5-fold the upper limit of normal), renal dysfunction (serum creatinine ≥115 µmol/L, or glomerular filtration rate [GFR] <60 mL/min/1.73 m^2^), hyper- or hypothyroidism, acute infection, malignant tumor, or psychiatric disease were also excluded. All participants were asked to report their clinical information regarding diabetes duration, alcohol consumption, smoking status, and medications. This study was approved by the Ethics Committee of Shanghai Jiao Tong University Affiliated Sixth People’s Hospital. Informed consent was provided by all participants prior to enrollment.

### Anthropometric measurements

The body mass index (BMI) of each subject was calculated as weight in kilograms divided by the square of height in meters. Blood pressure was measured with a mercury sphygmomanometer after the subject had rested for at least 10 min. Waist circumference (W) was measured midway between the lowest rib and the iliac crest with the subject in the standing position.

### Laboratory measurements

Fasting blood samples were collected from each subject after 10 h of overnight fasting for measurement of fasting plasma glucose (FPG), glycated hemoglobin A_1c_ (HbA_1c_), fasting C-peptide (FCP), total cholesterol (TC), triglyceride (TG), high-density lipoprotein cholesterol (HDL-c), low-density lipoprotein cholesterol (LDL-c), C-reactive protein (CRP), calcium (Ca), and phosphorus (P) levels. Approximately 2 h after eating breakfast, 2-hour plasma glucose (2hPG) and 2-hour C-peptide (2hCP) levels were assessed. Standard laboratory measurements were performed as described previously [13]. A Kainos sandwich enzyme-linked immunosorbent assay kit (Kainos Laboratories Inc., Tokyo, Japan) was used to determine serum FGF23 levels, and the intra- and inter-assay coefficients of variations were 5.6 and 8.2%, respectively. Serum C-peptide levels were measured by an electrochemiluminescence immunoassay (Roche Diagnostics GmbH, Mannheim, Germany) using the Cobas e 601 analyzer. The HOMA Calculator v2.2.3 released by the University of Oxford was used to estimate the insulin resistance index (homeostasis model assessment 2 of insulin resistance [HOMA2-IR]) using the FPG and FCP levels [16]. The GFR was accurately determined by technetium-99m diethylene triamine pentaacetic acid (Tc^99m^-DTPA) clearance.

### Ultrasonography measurements

All participants underwent color Doppler ultrasound examinations of lower limb arteries using an Acuson Sequoia 512 scanner (Siemens Medical Solutions, Mountain View, CA, USA) equipped with a 5–13 MHz linear array transducer. Ultrasound examination included measurement of atherosclerotic plaque and femoral IMT (F-IMT). Seven arteries in each lower limb, including the femoral artery, deep femoral artery, superficial femoral artery, popliteal artery, anterior tibial artery, posterior tibial artery, and peroneal artery, were checked for atherosclerotic plaque. IMT was defined as the distance between the leading edge of the lumen-intima echo and the leading edge of the media-adventitia echo [17]. F-IMT was defined as the mean value of IMTs of the bilateral femoral arteries [18].

### Diagnostic criteria

Diabetes was diagnosed based on the 2010 American Diabetes Association standards [19]. According to the Mannheim consensus [17], atherosclerotic plaque was defined as the presence of a focal structure encroaching into the arterial lumen at least 0.5 mm, or at least 50% greater than the thickness of the surrounding vessel wall, or an IMT ≥1.5 mm. LEAD was defined when atherosclerotic plaques were present in any of the lower limb artery segments listed above [20].

### Statistical analysis

Statistical analyses were performed using SPSS 17.0 software package (SPSS Inc., Chicago, IL, USA). All variables underwent a normality test. Normally distributed data and F-IMT data are presented as mean ± standard deviation (SD), and skewed data are expressed as the median with interquartile range. Categorical variables are expressed as a percentage (%). Inter-group comparisons of normally distributed data and skewed data were carried out by the unpaired Student’s *t* test and Mann–Whitney U test, respectively. The Chi square test was used for inter-group comparisons of categorical variables. Subgroup analysis was performed separately in men and women. Logistic regression analysis was performed to identify multiple factors associated with LEAD. Odd ratio (OR) was calculated to determine whether the relevant factors were risk factors for LEAD. Multiple linear regression analysis was conducted to identify factors independently correlated with the serum FGF23 levels. A two-tailed *P* value of <0.05 was considered indicative of a statistically significant difference.

## Results

### Clinical characteristics of the study participants

A total of 401 T2DM participants were enrolled in the present study [median age: 58 (48–64) years], including 201 subjects with LEAD and 200 subjects without LEAD. As shown in Table 1, compared with those without LEAD, subjects with LEAD were older and had higher systolic blood pressure (SBP), F-IMT, and ratio of anti-hypertensive therapy (all *P* < 0.01), along with a lower GFR (*P* < 0.01). In addition, subjects with LEAD also exhibited a longer diabetes duration than those without LEAD (*P* < 0.01). Other variables did not differ significantly between the two groups (all *P* > 0.05).

**Table 1:** Characteristics of the study participants

Variable	Total	Without LEAD	With LEAD
*n* (men/women)	401 (203/198)	200 (101/99)	201 (102/99)
Age (years)	58 (48–64)	50 (41–58)	62 (59–68)**
BMI (kg/m^2^)	25.59 ± 3.14	25.79 ± 3.27	25.38 ± 3.00
W (cm)	92.51 ± 9.51	91.61 ± 9.43	93.41 ± 9.52
SBP (mmHg)	130 (120–140)	125 (118–134)	130 (120–142)**
DBP (mmHg)	80 (70–85)	78 (70–82)	80 (70–85)
FPG (mmol/L)	7.72 (6.45–9.44)	7.78 (6.44–9.63)	7.65 (6.45–9.23)
2hPG (mmol/L)	13.10 ± 4.23	13.05 ± 4.47	13.15 ± 4.00
HbA_1c_ (%)	8.7 ± 1.9	8.8 ± 2.0	8.7 ± 1.8
HbA_1c_ (mmol/mol)	72 ± 21	72 ± 22	72 ± 20
FCP (ng/mL)	1.84 ± 0.87	1.81 ± 0.84	1.87 ± 0.89
2hCP (ng/mL)	4.13 (2.53–6.48)	4.13 (2.39–6.53)	4.09 (2.55–6.40)
HOMA2-IR	1.57 ± 0.75	1.56 ± 0.76	1.59 ± 0.75
TC (mmol/L)	4.74 ± 1.01	4.83 ± 0.86	4.66 ± 1.14
TG (mmol/L)	1.47 (1.02–2.24)	1.54 (1.02–2.39)	1.36 (1.01–2.12)
HDL-c (mmol/L)	1.05 (0.90–1.20)	1.06 (0.90–1.22)	1.05 (0.90–1.18)
LDL-c (mmol/L)	2.86 ± 0.81	2.92 ± 0.72	2.80 ± 0.90
CRP (mg/L)	0.85 (0.40–1.85)	0.89 (0.42–1.78)	0.83 (0.37–1.92)
GFR (mL/min/1.73 m^2^)	99.01 ± 20.37	105.43 ± 20.91	92.63 ± 17.69**
Ca (mmol/L)	2.35 ± 0.10	2.35 ± 0.10	2.34 ± 0.10
P (mmol/L)	1.27 ± 0.17	1.29 ± 0.17	1.26 ± 0.16
F-IMT (mm)	0.79 ± 0.11	0.72 ± 0.12	0.85 ± 0.01**
Current smoker, *n* (%)	103 (25.69)	45 (22.50)	58 (28.86)
Current drinker, *n* (%)	58 (14.46)	27 (13.50)	31 (15.42)
Anti-hypertensive therapy, *n* (%)	173 (43.14)	72 (36.00)	101 (50.25)**
Lipid-lowing therapy, *n* (%)	66 (16.46)	33 (16.50)	33 (16.42)
Diabetes duration (years)	8.00 (4.00–12.00)	6.00 (3.00–10.00)	10.00 (6.00–15.00)**

Data are expressed as mean ± SD, median (interquartile range), or *n* (%)

*BMI* body mass index, *W* waist circumference, *SBP* systolic blood pressure, *DBP* diastolic blood pressure, *FPG* fasting plasma glucose, *2hPG* 2-hour plasma glucose, *HbA*_*1c*_ glycated hemoglobin A_1c_, *FCP* fasting C-peptide, *2hCP* 2-hour C-peptide, *HOMA2*-*IR* homeostasis model assessment 2 of insulin resistance, *TC* total cholesterol, *TG* triglyceride, *HDL*-*c* high-density lipoprotein cholesterol, *LDL*-*c* low-density lipoprotein cholesterol, *CRP* C-reactive protein, *GFR* glomerular filtration rate, *Ca* calcium, *P* phosphorus, *F*-*IMT* femoral intima-media thickness

** *P* < 0.01 versus without LEAD

### Comparison of serum FGF23 levels

**Fig. 1:**
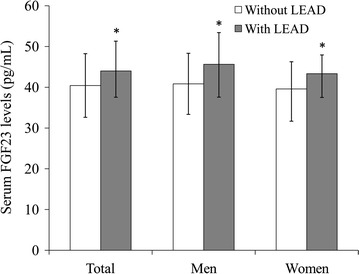
Comparison of serum FGF23 levels between diabetes patients with and without LEAD. Data are shown as median with 25th and 75th percentiles. *P < 0.05 versus without LEAD

Among the entire study population, the median (interquartile range) serum FGF23 levels were 42.08 (35.59–49.17) pg/mL. There was no significant gender difference in serum FGF23 levels (44.00 [35.83–49.58] pg/mL in men versus 41.67 [34.17–47.92] pg/mL in women, *P* > 0.05). Subjects with LEAD had significantly higher serum FGF23 levels compared with those without LEAD (44.00 [37.54–51.30] pg/mL versus 40.42 [32.61–48.23] pg/mL, *P* < 0.001). Subgroup analysis confirmed the significant difference in serum FGF23 levels between subjects with and without LEAD separately in men and women (45.63 [37.57–53.41] pg/mL versus 40.83 [33.33–48.33] pg/mL in men, *P* = 0.001; 43.33 [37.50–47.92] pg/mL versus 39.58 [31.67–46.25] pg/mL in women, *P* = 0.009; Fig. 1).

### Multiple factors related to LEAD

In multiple logistic analysis, the presence of LEAD was defined as the dependent variable and age, gender, BMI, W, SBP, diastolic blood pressure (DBP), HbA_1c_, HOMA2-IR, TC, TG, HDL-c, LDL-c, CRP, GFR, Ca, P, serum FGF23 levels, diabetes duration, current smoking, current alcohol use, anti-hypertensive therapy, and lipid-lowing therapy were defined as independent variables. The analysis identified the serum FGF23 levels (OR 1.039, 95% confidence interval [CI] 1.012–1.067, *P* = 0.004) as an independent and positive factor for LEAD, along with age (OR 1.180, 95% CI 1.137–1.225, *P* < 0.001), diabetes duration (OR 1.103, 95% CI 1.043–1.167, *P* = 0.001), and current smoking (OR 2.871, 95% CI 1.497–5.507, *P* = 0.002; Table 2).

**Table 2:** Independent factors for LEAD identified by multivariate logistic regression analysis

Variable	*β*	SE	OR (95% CI)	*P*
Age	0.165	0.019	1.180 (1.137–1.225)	<0.001
FGF23	0.039	0.014	1.039 (1.012–1.067)	0.004
Diabetes duration	0.098	0.029	1.103 (1.043–1.167)	0.001
Current smoking	1.055	0.332	2.871 (1.497–5.507)	0.002

Independent variables originally included: age, gender, BMI, W, SBP, DBP, HbA_1c_, HOMA2-IR, TC, TG, HDL-c, LDL-c, CRP, GFR, Ca, P, FGF23, diabetes duration, current smoking, current alcohol use, anti-hypertensive therapy, and lipid-lowing therapy

*BMI* body mass index, *W* waist circumference, *SBP* systolic blood pressure, *DBP* diastolic blood pressure, *HbA*_*1c*_ glycated hemoglobin A_1c_, *HOMA2*-*IR* homeostasis model assessment 2 of insulin resistance, *TC* total cholesterol, *TG* triglyceride, *HDL*-*c* high-density lipoprotein cholesterol, *LDL*-*c* low-density lipoprotein cholesterol, *CRP* C-reactive protein, *GFR* glomerular filtration rate, *Ca* calcium, *P* phosphorus, *FGF23* fibroblast growth factor 23

### Multiple stepwise regression analysis of serum FGF23 levels

Spearman correlation analysis showed that serum FGF23 levels were positively associated with age, FCP, 2hCP, W, and F-IMT (all *P* < 0.05). To further identify factors independently affecting serum FGF23 levels, a multiple liner regression model was used. The dependent variable was serum FGF23 levels, and the independent variables were age, FCP, 2hCP, W, and F-IMT. The analysis revealed that W (standardized *β* = 0.119, *P* = 0.016) and F-IMT (standardized *β* = 0.175, *P* < 0.001) were independently and positively correlated with serum FGF23 levels. Furthermore, these relationships remained significant after additional adjustment for gender and factors potentially affecting serum FGF23 levels (Ca, P, and GFR), respectively (both *P* < 0.05; Table 3).

**Table 3:** Multivariate regression analyses of factors associated with serum FGF23 levels

Variable	Standardized *β*	*t*	*P*
Model 1			
W	0.119	2.411	0.016
F-IMT	0.175	3.582	<0.001
Model 2 (adjusted for gender)			
W	0.115	2.335	0.020
F-IMT	0.184	3.748	<0.001
Model 3 (adjusted for Ca, P, and GFR)			
W	0.134	2.717	0.007
F-IMT	0.184	3.485	0.001

Original variables included: age, FCP, 2hCP, W, and F-IMT

*FCP* fasting C-peptide, *2hCP* 2-hour C-peptide, *W* waist circumference, *F*-*IMT* femoral intima-media thickness

## Discussion

The present study demonstrated that serum FGF23 levels were significantly increased in Chinese T2DM patients with LEAD. Our analyses showed that increased serum FGF23 levels were an independent risk factor for LEAD and positively associated with F-IMT. Additionally, a positive correlation between serum FGF23 levels and W was observed.

Accumulating clinical evidence indicates a link between serum FGF23 levels and atherosclerosis [21–25], which is also supported by our previous findings. We previously demonstrated that serum FGF23 levels were not only independently and positively associated with the presence of CAD, but also increased with the cumulative number of stenotic vessels [11]. Moreover, a correlation between serum FGF23 levels and carotid IMT has been reported previously by our study team in a Chinese population without diabetes, even in individuals with normal glucose tolerance [12, 13]. In the present study, serum FGF23 levels were also shown to be independently and positively associated with F-IMT. Because IMT can serve as an indicator of early atherosclerosis, our findings suggest the application of serum FGF23 levels for indicating clinical atherosclerosis in the early stage.

LEAD is one of the clinical manifestations of atherosclerosis that involves the lower extremity arteries. Recently, some studies have explored the relationship between serum FGF23 levels and LEAD. Biscetti et al. [26] diagnosed PAD according to the ankle brachial index and defined its severity according to the Fontaine’s staging system. In 976 Italian patients with T2DM, they found that serum FGF23 levels were independently and positively correlated with the presence and severity of PAD, suggesting that elevated serum FGF23 levels were an important risk factor for PAD (including LEAD). Another study (through diagnostic algorithms and physician adjudication) conducted on community-based cohorts of 659 American women also observed that subjects in the highest FGF23 tertile had a nearly twofold greater risk for PAD compared with those in the lowest tertile [27]. Different from the studies described above, the present study evaluated the arterial wall and plaque deposition directly via color Doppler ultrasound and made the diagnosis of LEAD based on these measurements [28]. The results suggested that elevated serum levels of FGF23 in T2DM patients could help indicate the presence of LEAD.

However, despite the relationships between elevated serum FGF23 levels and increased risk of LEAD demonstrated in an American follow-up study of 9.8 years, these relationships attenuated to be no longer statistically significant after additional adjustment for indexes of kidney function (estimated GFR and urinary albumin to creatinine ratio) [29]. Inconsistently, the present study showed that, after adjustment for GFR (accurately determined by Tc^99m^-DTPA clearance) as well as for Ca and P, the association between serum FGF23 levels and the presence of LEAD remained statistically significant. Recent studies suggested that higher levels of FGF23 are associated with diabetic nephropathy [30, 31]. In consideration of the influence of kidney function on serum FGF23 levels, the present study only enrolled subjects with normal kidney function (serum creatinine <115 µmol/L and GFR **≥**60 mL/min/1.73 m^2^). Studies with larger sample sizes are needed to confirm whether this relationship is influenced by kidney function.

Usually, abdominal obesity is indicated by the simple index W or the more accurate index, visceral fat area. The association between increased circulating FGF23 levels and W has been demonstrated in the Framingham Heart Study, the Osteoporotic Fractures in Men Study, and the Prospective Investigation of the Vasculature in Uppsala Seniors study [32, 33]. Previously, we reported a relationship between the serum FGF23 levels and visceral fat area [14]. Consistent with these previous findings, serum FGF23 levels were independently and positively related to W in the present study, reminding that abdominal obesity should be taken into consideration when exploring abnormal serum FGF23 levels.

In addition, previous studies reported that age, diabetes duration, and current smoker status are risk factors for LEAD [4, 34]. Consistent with these studies, we also found that these traditional risk factors were positively and independently associated with the presence of LEAD in T2DM patients.

The underlying mechanism via which FGF23 may contribute to LEAD in patients with diabetes remained unclear. Basic studies have suggested that the effects of FGF23 on vascular calcification and endothelial dysfunction might explain the mechanism to some extent. Richter et al. [7] demonstrated that in human coronary artery endothelial cells, FGF23 promoted oxidative stress, which induced nitric oxide release, and its stimulating effects on reactive oxygen species production were counterbalanced by increased reactive oxygen species degradation and suppressed the nitric oxide bioavailability, thus leading to endothelial dysfunction. Research in an animal model detected increases in FGF23 expression at both the mRNA and protein levels in rats with vascular calcification, suggesting that FGF23 might enhance the vascular calcification [8]. In addition, current basic evidence links endothelial dysfunction to diabetes through demonstration of impaired endothelial-dependent vasodilation [35]. Due to multiple homeostatic imbalances occurring with hyperglycemia in diabetes, endothelial dysfunction might be induced by multifactorial etiologies, of which altered FGF23 might be one. The elevation in circulating FGF23 levels, which was also observed in diabetic animal models [36, 37], might aggravate the existing endothelial dysfunction and stimulate vascular calcification, ultimately leading to atherosclerosis in diabetes.

The present study was limited by the cross-sectional design and relatively small sample size, which made it difficult to clarify the causal relationship between increased serum FGF23 levels and the presence of LEAD. Secondly, the study population was restricted to patients with T2DM. Hence, further prospective studies are warranted to confirm and generalize the present findings in a larger population, including those without diabetes. Thirdly, the present study only investigated the relationship between serum FGF23 levels and LEAD. Future research is needed to explore the associations between serum FGF23 levels and other complications of diabetes, such as diabetic retinopathy and nephropathy. In addition, the present study design did not detect ankle brachial index, and the corresponding data are needed to further analyze the association of serum FGF23 levels with the early stage of LEAD in future studies.

## Conclusions

As a conclusion, in Chinese patients with T2DM, serum FGF23 levels were significantly increased with the presence of LEAD, independent of other cardiovascular risk factors. Therefore, elevated serum FGF23 levels were associated with the presence of LEAD in this population.

## Abbreviations

BMI: body mass index
Ca: calcium
CRP: C-reactive protein
DBP: diastolic blood pressure
FCP: fasting C-peptide
FGF: fibroblast growth factor
F-IMT: femoral intima-media thickness
FPG: fasting plasma glucose
GFR: glomerular filtration rate
HbA_1c_: glycated hemoglobin A_1c_
HDL-c: high-density lipoprotein cholesterol
HOMA2-IR: homeostasis model assessment 2 of insulin resistance
IMT: intima-media thickness
LDL-c: low-density lipoprotein cholesterol
LEAD: lower extremity atherosclerotic disease
P: phosphorus
PAD: peripheral arterial disease
SBP: systolic blood pressure
T2DM: type 2 diabetes mellitus
TC: total cholesterol
Tc^99m^-DTPA: technetium-99m diethylene triamine pentaacetic acid
TG: triglyceride
W: waist circumference
2hCP: 2-hour C-peptide
2hPG: 2-hour plasma glucose
